# Effects of maternal arsenic exposure on birth outcomes using harmonized data across three birth cohorts

**DOI:** 10.1007/s13530-025-00292-6

**Published:** 2026-01-29

**Authors:** Zlatan Feric, Daniel Beene, Antonio J. Signes-Pastor, Deborah J. Watkins, Griffith Gao, Margaret R. Karagas, Debra A. MacKenzie, David R. Kaeli, Justin Manjourides

**Affiliations:** 1 Department of Electrical and Computer Engineering, Northeastern University, Boston, MA, USA; 2 Department of Epidemiology, Johns Hopkins University Bloomberg School of Public Health, Baltimore, MD, USA; 3 Instituto de Investigación Sanitaria y Biomédica de Alicante (ISABIAL), Alicante, Spain; 4 Departamento de Salud Pública, Historia de La Ciencia y Ginecología, Unidad de Epidemiología de La Nutrición, Universidad Miguel Hernández (UMH), Alicante, Spain; 5 CIBER Epidemiología y Salud Pública (CIBERESP), Instituto de Salud Carlos III, Madrid, Spain; 6 Department of Environmental Health Sciences, University of Michigan School of Public Health, Ann Arbor, MI, USA; 7 Department of Epidemiology, Dartmouth Geisel School of Medicine, Hanover, NH, USA; 8 College of Pharmacy, Community Environmental Health Program, University of New Mexico Health Sciences Center, Albuquerque, NM, USA; 9 Department of Public Health and Health Sciences, Northeastern University, Boston, MA, USA

**Keywords:** Data harmonization, Cohort studies, Arsenic exposure, Birth outcomes

## Abstract

**Objectives:**

To assess the association between urinary arsenic concentrations and birth outcomes by harmonizing data from three independent birth cohorts.

**Methods:**

We harmonized and analyzed data from the Navajo Birth Cohort Study (NBCS), the New Hampshire Birth Cohort Study (NHBCS), and the PROTECT Center study based on Puerto Rico. Birth outcomes of interest included birth weight, head circumference, birth length, gestational age at delivery, incidence of preterm birth, and size for gestational age. Urinary arsenic concentrations were used as the primary exposure metric. Harmonization involved aligning variable formats, adjusting for differences in laboratory methods, and excluding incompatible covariates, such as income and race.

**Results:**

Harmonization increased the total sample size (*N* = 3222) across cohorts. However, pooled analyses did not consistently demonstrate increased statistical power. Effect estimates for arsenic exposure were attenuated in some cases, and confidence intervals remained wide or even expanded relative to individual cohort analyses. Differences in biospecimen collection and laboratory assay methods required cohort-specific adjustments. Due to missing arsenic speciation data, the PROTECT cohort was excluded from two exposure models. Variability across cohorts limited the interpretability and precision of pooled estimates despite harmonization efforts.

**Conclusions:**

While harmonizing data across multiple cohorts increased the sample size, it did not necessarily enhance statistical power or strengthen observed associations. Differences in data collection, laboratory methods, and available covariates posed significant challenges. These findings underscore the need for caution when interpreting pooled results from heterogeneous sources and highlight the importance of prospective planning for data harmonization in multi-cohort studies.

## Introduction

Data harmonization is an emerging theme in environmental health science literature and funding mandates aimed at uncovering previously hidden associations between diverse environmental exposures and health outcomes [1]. Posited benefits of data harmonization include improved data quality, increased collaboration, and increased statistical power of resultant analyses [2–4].

In this study, we harmonized data from three birth cohorts to assess the effect of maternal arsenic exposure on birth outcomes. This work leverages data collected by three independent birth cohorts that have collaborated on this harmonization effort: 1) the Navajo Birth Cohort Study (NBCS) based on the Southwestern United States led by the University of New Mexico; 2) the New Hampshire Birth Cohort Study (NHBCS) led by Dartmouth College; and 3) the PROTECT Center based on Puerto Rico and led by Northeastern University. These three cohorts were selected for this collaborative research project as they each participate in the broader National Institute of Environmental Health Sciences Superfund Research Program, and thus collect data on similar environmental contaminants and outcomes.

The enhanced analyses facilitated by combining data across multiple cohorts are largely dependent on the quality and interoperability of individual data sets. Feasibility of harmonization must be assessed by comparing data dictionaries, standard operating procedures, and questionnaires across studies [5, 6]. For example, laboratory-based methods for detecting concentrations of particular analytes in biological media such as urine or blood will have different limits of detection (LOD) or measurement standards that must be considered when determining the interoperability across populations and time frames. Similarly, questionnaires that categorize contextual variables such as household income or education levels of study participants may not easily translate across populations.

Arsenic is a metalloid present in environmental media in both organic and inorganic forms. People are frequently exposed to organic arsenic compounds through common food items, such as rice and seafood, and to inorganic arsenic through consumption of contaminated drinking water, inhalation of metal-bearing dusts, and via medications [7, 8]. The toxicity of inorganic arsenic is well-documented, with evidence of associations between prenatal exposure and low birth weight [8, 9]. Specifically, arsenic is a teratogen that readily passes the placental barrier; often arsenic concentrations are similar in both maternal and cord blood samples [10]. In addition, because the metalloid targets enzyme reactions throughout the body, chronic arsenic exposure is associated with multiple endpoints—particularly dermal effects, such as hyperkeratosis and hyperpigmentation. It is also a known carcinogen and has been linked to cancers of the skin, bladder, and lung [8].

Inorganic arsenic may be distributed through the environment heterogeneously via water and air pathways and is often introduced into these media by anthropogenic activities, such as hardrock mining, oil and natural gas production, waste disposal, and other forms of resource and energy production [11]. More than 70% of Superfund sites contain observable levels of environmental arsenic and other deleterious metals [8]. While the prevalence of arsenic in drinking water sources is well-established [12–14], the geographic diversity and environmental mobility of environmental arsenic concentrations in water warrant continued investigation. Furthermore, the role of arsenic-bearing dust on health outcomes remains understudied, though the association between potential inhalation and urinary arsenic concentrations has been demonstrated [7].

In this work, we combined three distinct cohorts to investigate the association between urinary arsenic concentrations and several birth outcomes while demonstrating that harmonization of data across heterogeneous populations may not always lead to increased statistical power and may obscure meaningful subgroup effects.

## Materials and methods

### Cohorts and study participants

In the PROTECT prospective birth cohort, pregnant women were recruited from prenatal clinics and hospitals in northern Puerto Rico for participation from 2010 through 2022. Women were recruited at approximately 14 ± 2 weeks of gestation and were eligible if they were between 18 and 40 years, lived in the Northern karst region of Puerto Rico, did not use oral contraceptives 3 months prior to pregnancy, did not use in vitro fertilization to become pregnant, and did not have known medical/obstetric complications [15, 16]. Residents of Puerto Rico are disproportionately exposed to higher levels of metals and metalloids than those in the continental U.S. [17], due in large part to the abundance of hazardous waste sites across the island, many of which are situated in unlined landfills located above the karst aquifer [16].

The New Hampshire Birth Cohort Study (NHBCS) is led by investigators at The Geisel School of Medicine at Dartmouth College and in 2009 began recruiting rural New Hampshire and Vermont residents between the ages of 18 and 45 with a confirmed pregnancy, and initially focused on those who relied on private unregulated wells as their primary source of drinking water [18]. It is estimated that approximately 10% of all private unregulated wells in New Hampshire contain arsenic concentrations higher than the maximum contaminant level (MCL) of 10 μg/L and even more above the New Hampshire MCL of 5 μg/L [8, 9].

The Navajo Birth Cohort Study (NBCS) is operated out of the University of New Mexico Community Environmental Health Program [19]. NBCS participants are Navajo women aged 14–45 years with a confirmed pregnancy at the time of recruitment, beginning in 2013. Biomonitoring data (blood, serum, and urine samples), home environmental assessments, and demographic/behavioral data are collected for consenting participants. The Navajo Nation is the largest tribal reservation in the American Southwest spanning nearly 30,000 square miles across New Mexico, Arizona, and Utah. Situated on the Colorado Plateau, the lands of the Navajo Nation are home to significant extractive activities, including hard rock mining and oil and natural gas production. Navajo residents are exposed to environmental toxicants such as metals and metalloids stemming largely from an intractable legacy of uranium mining that has been characterized by inadequate mine remediation and spotty federal oversight. The pathways of environmental exposure are numerous, as mine waste is released through overland flow of surface water, has seeped into groundwater sources, and Aeolian (wind-blown) processes re-suspend metal-bearing dust. Consumption of contaminated water and the presence of household dust are significant sources of exposure [7].

### Biospecimen sample collection and analysis

Spot urinary samples were collected during pregnancy from birthing parents in all three cohorts. All samples were processed following best practices and urinary arsenic species concentrations including inorganic arsenic, dimethylated arsenic (DMA), monomethylarsonic acid (MMA), and arsenobetaine were measured with inductively coupled plasma-dynamic reaction cell–mass spectrometry (ICP–MS) at the CDC NCEH’s DLS with the exception of NHBCS samples, which were analyzed using anion exchange chromatography inductively coupled plasma mass spectrometry (HPLC–ICP–MS) by the Trace Element Analysis Core at Dartmouth College [20–26].

NHBCS calculated the limits of detection as the mean of the blank concentrations plus three times their standard deviation multiplied by the dilution factor. For all cohorts, concentrations below the lower limit of detection (LLOD) were imputed as the instrument’s LLOD divided by the square root of $2\left(\frac{\text{LLOD}}{\sqrt{2}}\right)$ [25]. Table 1 shows summary statistics of the range of LLODs as well as the number of observations below the LLOD for each analyte by cohort.

### Harmonization

Given the limited availability of tools for harmonizing environmental health data, we developed a customized, secure online harmonization framework to facilitate this process. The full details of this harmonization procedure, including the extract-transform-load (ETL) process and mappings are described in Feric et al., 2021 [5]. Briefly, each site utilized the data harmonization application to upload their cohort-specific data to our secure app, which evaluated the data against the specified format and ranges. Next, the back end of the application utilized data ETL procedures for each cohort which transformed the cohort-specific data into the common data model (e.g., transformation of units and data layout) which could then be recombined into one pooled data set for analysis. Finally, our analysis was carried out through a secure Jupyter notebook which was hosted on the app and provided direct access to the common data model in the database.

### Urinary arsenic and birth outcomes

Both UTAS and arsenic species were harmonized across all three cohorts where available (only UTAS was collected for the PROTECT cohort), ensuring that concentrations have the same units, μg/L. For both the PROTECT and NHBCS cohorts, only urine samples collected in the third trimester (after 27 gestational weeks) were included. However, all maternal urine samples from NBCS were included in the analyses, because participant recruitment occurred at any point during pregnancy, and limiting samples to the third trimester would significantly reduce the sample size.

To estimate the gestational age of infants, we utilized either fundal height or self-reported last menstrual period and subsequently classified the birth as either preterm (before 37 weeks) or term (37 weeks or later). Infants’ sex, weight (grams), head circumference (cm), and length (cm) were collected from medical records in accordance with appropriate informed consent from all participants. We further derived binary variables for infants who were classified as small for gestational age (SGA) or large for gestational age (LGA) based on international birth weight standards [27].

### Covariates

Harmonized covariates include biophysical, behavioral, and socioeconomic information about each participant collected as part of each cohort’s approved study design and participant informed consent. The following data were collected from medical record abstractions and self-report questionnaires: maternal age (in years based on reported birth date), race and ethnicity, pre-pregnancy body mass index (BMI, kg/m^2^), weight gain during pregnancy (kg), delivery type, pregnancy complications, folic acid supplementation, fish/seafood consumption (yes/no), parity, smoking history (never/current/former smoker), and smoking during pregnancy. Ceremonial tobacco use in the NBCS cohort was not considered to be ‘current smoker’. Urinary dilution was characterized using either specific gravity or creatinine concentrations and is described further in the following section. Maternal education level is a categorical variable with conflicting variable levels across cohorts and thus was normalized to the following levels: less than 11th grade; high school graduate or equivalent; junior college graduate; college graduate; any post-graduate schooling.

### Urinary dilution adjustment

We standardized arsenic concentrations for urinary dilution using measures of either specific gravity (cohorts NHBCS, PROTECT) or creatinine (cohort NBCS). We utilized a modified version of the method described by [28], which allowed us to obtain comparable, standardized measurements using either creatinine or specific gravity as previously described by Kuiper et al. [29]. This method was chosen to enable combining arsenic data across cohorts that had measured different urinary dilution indicators. We did not utilize a covariate-adjusted method [29, 30] as this drastically reduced our sample size due to missing data. For cohorts with specific gravity measurements, the corrected arsenic concentration was calculated as

$$As_{corr_{SG}}=As_{obs}\times \frac{\left(SG_{\text{median}}-1\right)}{\left(SG_{obs}-1\right)}$$

where $As_{corr_{SG}}$ is the specific gravity-corrected arsenic concentration, *As*_*obs*_ is the observed arsenic concentration, *SG*_*median*_ is the median of specific gravity values in the cohort-specific study sample, and *SG*_*obs*_ is the observed specific gravity value. Similarly, for cohorts with urinary creatinine measurements, the corrected arsenic concentration was calculated using the following equation:

$$As_{corr_{Cr}}=As_{obs}\times \frac{Cr_{\text{median}}}{Cr_{obs}}$$

where $As_{corr_{Cr}}$ is the creatinine-corrected arsenic concentration, *As*_*obs*_ is the observed arsenic concentration, *Cr*_*median*_ is the median of creatinine concentrations in the cohort-specific study sample, and *Cr*_*obs*_ is the observed urinary creatinine concentration.

### Statistical analysis

Descriptive analyses of participants’ characteristics were calculated. Urine metal concentrations were natural logarithm transformed to reduce right skewness before statistical analyses. To evaluate residual between-cohort heterogeneity after harmonization, we applied the Anderson–Darling Test [31] for equality of distributions to each harmonized variable. This nonparametric test is sensitive to differences in both central tendency and distribution tails. Significant heterogeneity across cohorts could be consistent with differing recruitment and measurement procedures underscoring the importance of cohort adjustment in downstream analyses.

To account for skewed distributions of urinary arsenic concentrations and to observe the central tendency of the data set, we calculated the geometric mean (*gMean*) and geometric standard deviation (*gSD*). We also computed *gMean* and *gSD* urinary arsenic concentrations from National Health and Nutrition Examination Survey (NHANES) cycles 2011–2012, 2013–2014, 2015–2016, and 2017–2018 among women aged 14–45 as a benchmark for comparison. These were derived using 8-year sample weights to account for the complex survey design per CDC recommendations [32].

Linear regression analyses between continuous outcomes of interest (dependent variable) and maternal total urinary arsenic concentrations (independent variable, adjusted for maternal level of education, smoking during pregnancy, maternal BMI, parity, and infant sex) were performed (Model 1.1). We also conducted logistic regression analyses between binary outcomes of interest (dependent variables) and maternal total urinary arsenic concentrations (independent variables, adjusted for maternal level of education, smoking during pregnancy, maternal BMI, parity, and infants’ sex) (Model 1.2). We excluded participants with missing values for necessary covariates for each aforementioned analysis.

To assess the impact of the harmonization on statistical efficiency, confidence intervals were calculated for effect sizes estimated for individual cohorts and the models fitted to the harmonized data.

Sensitivity analyses were conducted by restricting models to those participants with arsenic speciation data. We fit linear regression models to estimate associations between continuous outcomes of interest and maternal summation of urinary arsenic concentrations (∑ *As* = *iAs* + *DMA* + *MMA*) while adjusting for maternal level of education, smoking during pregnancy, maternal BMI, parity, fish/seafood consumption and infant sex. (Model 2.1) The sum of arsenic species is a more accurate measure of exposure to inorganic arsenic (the known toxic form of arsenic) rather than total arsenic, as the latter may contain potentially non-toxic forms of arsenic, such as arsenobetaine. However, we used total arsenic in some instances, where cohorts did not list arsenic speciation, and as a sensitivity analysis. We also fit logistic regression models to estimate associations between binary outcomes of interest and maternal summation of urinary arsenic concentrations adjusting for maternal level of education, smoking during pregnancy, maternal BMI, parity, fish/seafood consumption and infant sex. (Model 2.2) Sensitivity analyses restricted to arsenic speciation data with arsenobetaine ≤ 1 μg/L were also performed (Models 3.1, 3.2). While our primary analyses focused on the covariate-adjusted effects on birth outcomes of the three different arsenic measures, we conducted additional exploratory analyses to examine potential effect modification by infant sex. Given the exploratory nature of these interaction analyses across 60 cohort–exposure–outcome pairs, we applied a Bonferroni correction factor resulting in a corresponding α = 0.05/60 = 0.0008.

We also investigated if significant effects could be generalized across all harmonized data or if they are more likely attributable to individual cohorts. We did this by evaluating (meta-analyses) the across-cohort differences in the effect of arsenic on each outcome. Both Cochran’s Q and *I*^2^ tests of heterogeneity were conducted on all exposure–outcome models. Cochran’s Q statistic assesses the likelihood that observed variability arises from heterogeneity between studies rather than from within study variation among individual subjects. The *I*^2^ statistic quantifies the proportion of total variability in effect estimates that is attributable to between-cohort heterogeneity rather than to chance. Differences of urinary arsenic concentrations between cohorts were evaluated using the Kruskal–Wallis test for total urinary arsenic across all three cohorts and Mann–Whitney *U* tests of arsenic species between NHBCS and NBCS populations. Significance of all tests was evaluated at an alpha level of 0.05.

## Results and discussion

### Cohort overview

Sample sizes available to describe characteristics and demographics of individual cohorts are presented in Table 2. At least partial information was available for a total of 3222 participants across the three cohorts. The majority of those participants, 2132, are observed through the NHBCS. PROTECT contributes data related to 570 participants, and NBCS contributes information from 520 participants. In general, the NHBCS has the most participant measures available, with the exception of creatinine, which was not collected by either NHBCS or PROTECT. These data contain over 90% completeness for all covariates and several outcomes. Outcomes with specific gravity of urine samples were only collected through NHBCS and PROTECT, while creatinine levels were collected only through NBCS. Arsenic speciation data were not available for PROTECT samples, and for only a subset of samples from NHBCS. The Anderson–Darling tests indicated significant distributional differences across cohorts for all harmonized variables (*p* < 0.05; Supplemental Table S1). These results confirm residual heterogeneity among cohorts despite standardized variable definitions.

Pregnant participants across the three cohorts had a mean (SD) age of 29.72 (5.4) years, and a mean prepregnancy BMI of 26.64 (6.3) (kg/m^2^) (Table 3). Maternal education and race varied most across cohorts (Table 4). Across all cohorts, births had a mean gestational age of 38.91 (1.9) weeks, mean length of 50.52 (3.1) cm and a mean weight of 3351.28 (543.2) grams (Table 3). Prepregnancy BMI was highest for the NBCS cohort, maternal age and birth weight were lowest for the PROTECT cohort, and gestational age and birth length did not vary greatly across the cohorts. Mann–Whitney tests indicate that total and speciated arsenic were statistically significantly different across cohorts. The *gMean* (*gSD*) of total arsenic across the three cohorts was calculated as 6.03 (3.2) μg/L, compared to a *gMean* (*gSD*) of 6.8 (1.04) μg/L in NHANES. Total urinary arsenic was highest in PROTECT, with a *gMean* of 9.66 μg/L (*gSD* = 2.44 μg/L), compared to NHBCS (*gMean* = 5.48 μg/L, *gSD* = 3.59 μg/L), and NBCS (*gMean* = 4.80 μg/L, *gSD* = 2.44 μg/L) (Tables 4, 5). Comparisons of speciated arsenic between NBCS and NHBCS similarly revealed significant differences in concentrations of arsenic III, arsenic IV, dimethylarsenic acid (DMA), and. monomethylarsonic acid (MMA), with higher *gMean* of each species concentration in the NBCS population but larger *gSD* in NHBCS (Table 3).

### Regression models

**Fig. 1:**
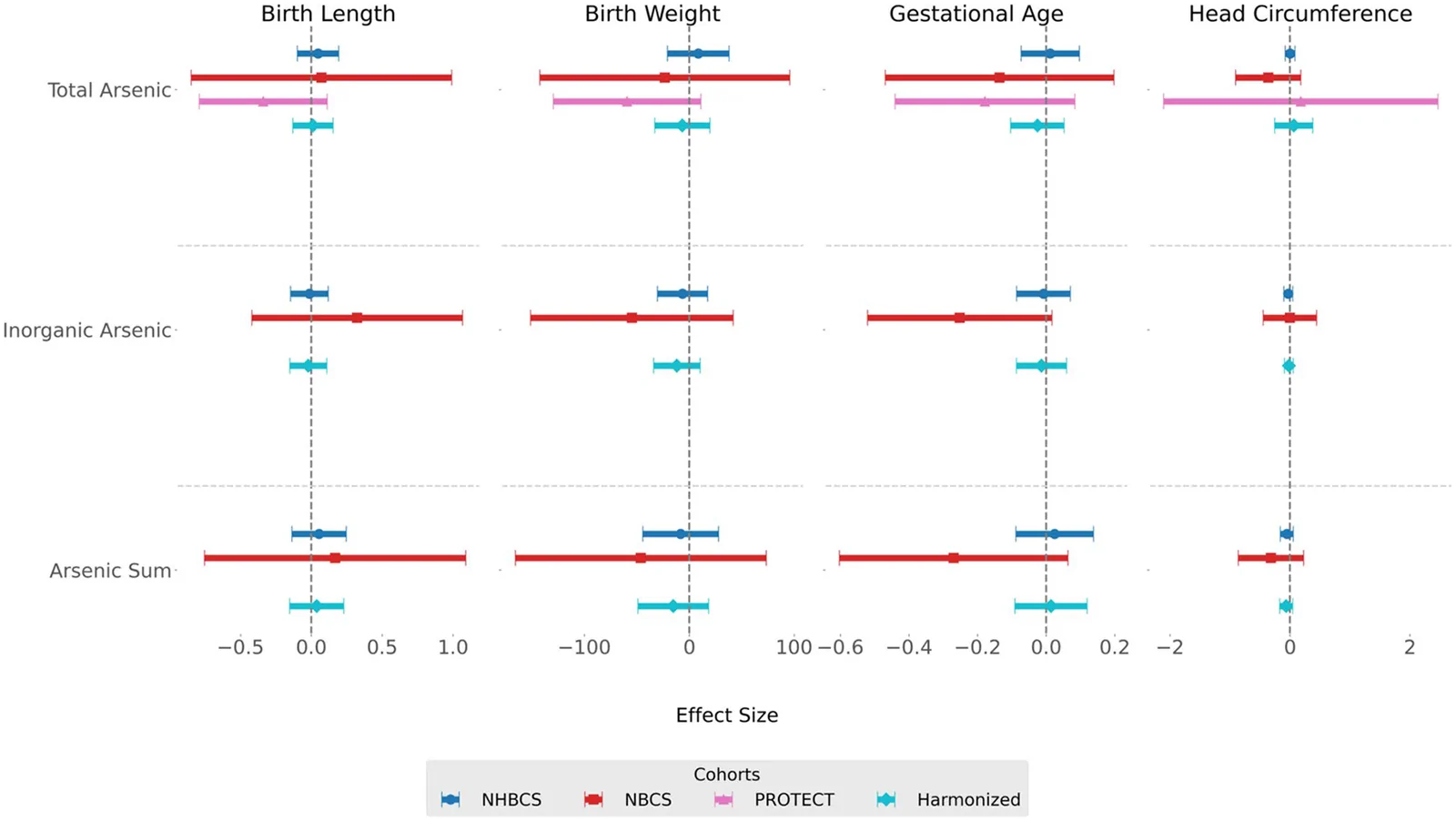
Associations between maternal urinary arsenic species concentrations and birth outcomes (models 1.1 and 2.1)

Results for primary regression models estimating associations between urinary arsenic concentrations and continuous birth outcomes and binary birth outcomes are presented in Tables 6 and 7, respectively. Across all exposure–outcome pairs, we did not observe any clear associations within individual cohorts or in analyses of the harmonized cohort data. We did observe, however, estimated effect sizes above and below zero across cohorts for the same exposure–outcome pair (e.g., total urinary arsenic and birth weight). That is, there were both positive and negative associations, although not at a significant level, of exposure on birth outcomes depending on the cohort analyzed. For example, total urinary arsenic was associated with a decrease in birth weight in PROTECT $\left(\hat{\beta }=-6.50\right)$, whereas it was associated with an increase in birth weight in NBCS $\left(\hat{\beta }=23.26\right)$ and NHBCS $\left(\hat{\beta }=8.64\right)$. These varying effect estimates are detailed in Table 6 and illustrated in Fig. 1.

In analyses examining potential effect modification by sex, we identified 7 exposure–outcome pairs with 95% confidence intervals not containing zero. After applying the Bonferroni correction for multiple testing, none of the interaction models reached statistical significance (Supplemental Table S2, Table 8).

Meta analysis shows that only the effect of the sum of inorganic arsenic on preterm birth measured using a logistic regression demonstrated significant cross-cohort heterogeneity (Q = 4.99, *p* = 0.03; *I*^2^ = 79.96%) (Table 9). Otherwise, we did not observe significant differences across cohorts in harmonized analyses.

Specific to our harmonization investigation and contrary to our expectations, average confidence interval widths did not necessarily decrease when combining cohorts for certain exposure–outcome pairs. For instance, as shown in Table 6, for the exposure–outcome pair involving UTAS and head circumference, the confidence width for the harmonized data set (CI width = 0.64) is larger when compared to NBCS (CI width = 0.16) alone, but smaller compared to PROTECT (4.58) and NHBCS (1.09) (Table 8).

## Discussion

### Exposure–outcome results

In this harmonized analysis across three U.S.-based birth cohorts (NHBCS, PROTECT, and NBCS), we found no consistent associations between maternal arsenic exposure measured as total, inorganic, or sum of arsenic species and key birth outcomes including weight, length, gestational age, or fetal growth indicators. However, these analyses had several limitations that may have influenced our ability to observe significant associations, as described below. These findings are generally consistent with prior literature, which has reported limited and sometimes inconsistent associations between low-level prenatal arsenic exposure and adverse birth outcomes. For instance, Bloom et al. concluded that epidemiologic evidence, particularly in low-exposure settings, remains insufficient to confirm causal associations with birth weight or gestational duration [33]. Similarly, Liu et al. observed effects primarily during the third trimester and only among female infants, highlighting both exposure timing measured via urinary arsenic and sex as potential modifiers [34].

While interaction analyses suggested potential sex-specific effects of a subset of exposure–outcome pairs, these findings are exploratory and require validation in independent cohorts. These hypothesis-generating findings warrant targeted investigation in future studies designed specifically to assess potential sex differences in the association between arsenic and birth outcomes.

Some cohort-specific trends in this study, such as the inverse associations with birth length observed in PROTECT, align with findings from higher exposure populations using different biological matrices to assess arsenic [35, 36], although the effect sizes in our sample were modest. Notably, Signes-Pastor et al. reported sex-specific associations between arsenic exposure measured in maternal toenails and birth outcomes in the NHBCS cohort [26].

Differences in exposure assessment, particularly the biological matrix used and the timing of sample collection during pregnancy, may partially explain discrepancies across studies. While our harmonized models did not stratify by sex to retain statistical power, this decision may have limited our ability to detect sex-specific associations.

### Statistical power implications on harmonization

**Fig. 2:**
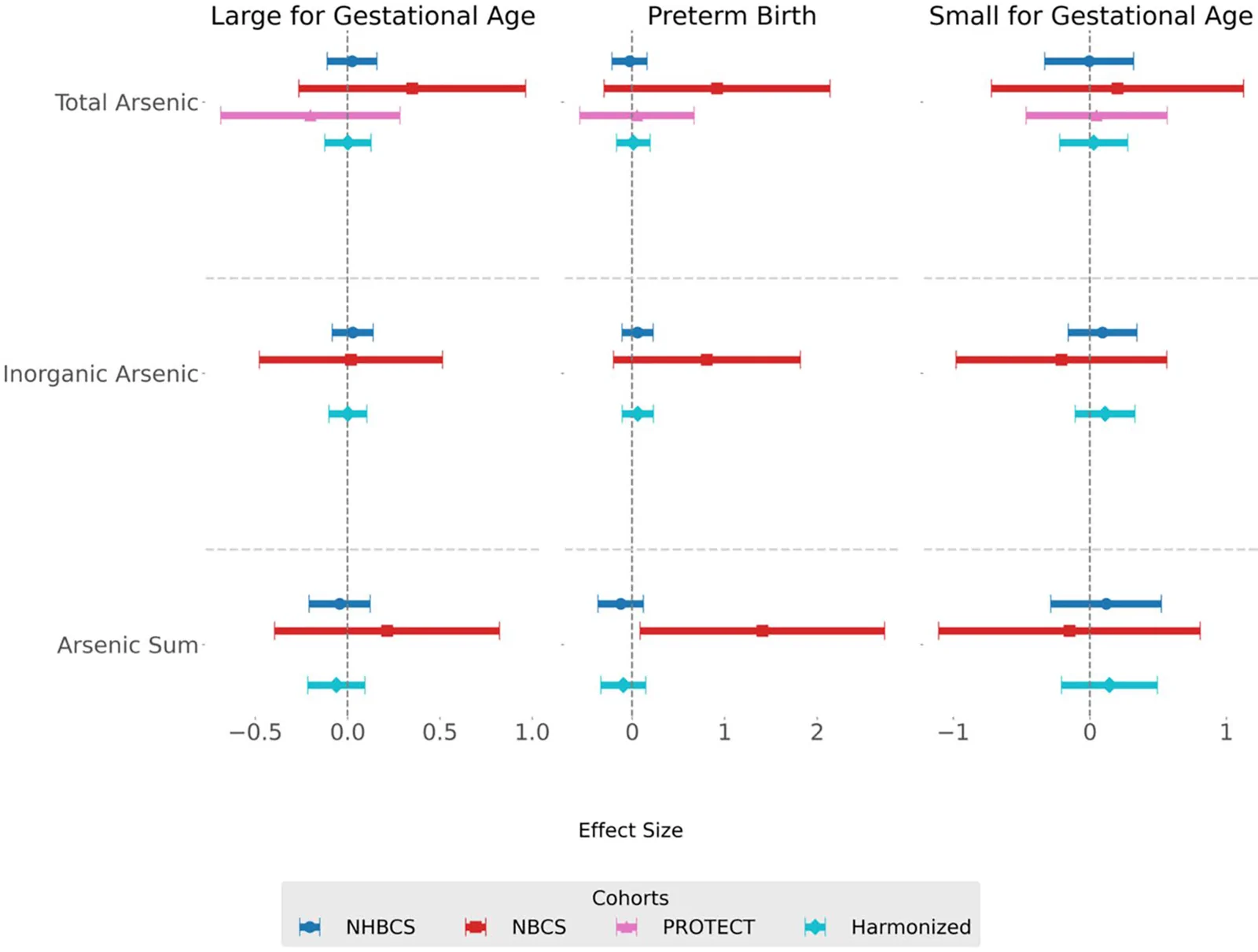
Associations between maternal urinary arsenic species concentrations and birth outcomes (models 1.2 and 2.2)

The harmonization of these three cohorts did not always lead to an expected improvement in statistical power [37]. This is evidenced by the confidence intervals that did not decrease in width and by some harmonized effect estimates from the regression models that did not show an association between exposures to arsenic and the birth outcome compared to estimates observed with higher precision in the individual cohorts. The increased widths of the confidence intervals are likely driven by between-cohort heterogeneity in outcome distributions contributing to larger overall standard errors in the outcome of interest, despite the increased sample sizes [38]. Individual cohort effect size estimates both above and below zero (e.g., the estimated effect of total arsenic on gestational age) resulted in attenuated measures of association between the exposures and outcomes in the harmonized data set (Figs. 1 and 2). The positive and negative effect estimates in exposure–outcome pairs across cohorts were likely averaged out in the regression of the harmonized data set and resulted in an overall diminished association between the exposure and outcome [39]. The combination of these two artifacts may have undermined the attempts to increase statistical power by simply combining multiple cohorts [40]. Further studies should identify conditions that may allow for a priori identification of cohorts that could be synergistically combined while not increasing the risk of false discoveries.

### Harmonization challenges

Different social/environmental contexts and resulting data collection protocols across cohorts rendered data harmonization intractable in some instances. Specifically, household income is a categorical variable that arranges participant income into levels that could not be disaggregated across cohorts. Aside from the harmonization challenge, it is potentially inappropriate to cross-tabulate income as an indicator of socioeconomic status across cohorts because of confounding regional, social/cultural, and economic contexts that relate to income [41, 42]. Race and ethnicity were also excluded as covariates, because each cohort lacks internal racial heterogeneity, so racial differences may be complicated by other structural (e.g., social/economic) drivers of health outcomes that cannot be adequately captured by the relatively low variability across and within each cohort. Because of these data harmonization challenges, we acknowledge that there is likely to be residual confounding by both unmeasured variables and variables not measured in all cohorts.

The PROTECT cohort does not report arsenic speciation, making it difficult to adequately distinguish potential pathways of exposure, such as coming from inhalation of dust, drinking water, or eating certain foods. Because consumption of certain foods is a common source of organic and inorganic arsenic that is rapidly digested, we anticipated that using food frequency questionnaires would allow us to determine whether a participants’ UTAS was associated with characteristics of their diet. However, we were unable to identify such an association. As such, seafood consumption was excluded as a covariate in our models and speciation is only examined in the NBCS and NHBCS cohorts.

Finally, though folic acid is implicated in a reduction of inorganic arsenic [43], inconsistent reporting of participants using supplements containing folic acid limited our ability to reliably include that variable in the harmonized data set.

## Conclusion

While data harmonization may often be beneficial by increasing the sample size, the procedure may not always result in increased statistical power or decreased confidence interval widths. Our harmonization study successfully pooled data across three cohorts and yet observed no significant associations between the exposures and outcomes. In fact, we observed wider confidence intervals corresponding to some exposure–outcome pairs, such as UTAS and head circumference, potentially highlighting limitations of harmonization.

The process of harmonization across birth cohorts has unique challenges. It is primarily confounded by each cohort’s unique social, demographic, and geographic contexts. Another challenge is the alignment of different data collection protocols and their timing, each of which is unique to the many different cohorts. Finally, the harmonization procedures may amplify the effects of missing data. As observed in this study, arsenic speciation data were limited to only two of the three cohorts (NHBCS and NBCS). This presents the opportunity for future investigation, such as exploring the development of models that can predict arsenic speciation variables in cohorts, where data are missing, by leveraging data from cohorts, where it is available.

## Supplementary Material

Supplemental Tables

**Supplementary Information** The online version contains supplementary material available at https://doi.org/10.1007/s13530-025-00292-6.

## Figures and Tables

**Table 1:** Summary statistics of limits of detection and number of observations below LOD by cohort

	NHBCS				PROTECT				NBCS			
	min	med	max	n BLOD	Min	med	max	n BLOD	min	med	max	n BLOD
UTAS	0.01	0.055	0.182	13 (0.84%)	0.3	0.3	3	1 (0.18%)	0.23	0.23	0.23	0 (0%)
UMMA	0.003	0.037	0.33	299 (23.73%)	–	–	–	–	0.2	0.2	0.2	279 (54.07%)
UDMA	0.017	0.05	0.39	24 (1.90%)	–	–	–	–	1.91	1.91	1.91	118 (22.87%)
UAS3	0.01	0.038	0.35	588 (27.91%)	–	–	–	–	0.12	0.12	0.12	235 (54.54%)
UAS5	0.02	0.067	0.35	909 (43.14%)	–	–	–	–	0.79	0.79	0.79	501 (97.09%)

**Table 2:** Number of observations across the three cohorts, categorized by variable type

		NHBCS	PROTECT	NBCS	Total (%)
Demographic	Sex	2132	570	520	3222 (99.38)
	Maternal Age	2151	569	451	3171 (97.81)
	Smoke During Pregnancy	1853	570	519	2942 (90.75)
	Maternal Race	2032	570	435	3037 (93.68)
	Maternal BMI	2078	538	298	2914 (89.88)
	Parity	2087	419	521	3027 (93.37)
	Education	1792	570	450	2812 (86.74)
Outcome	Birth Weight	1930	555	520	3005 (92.69)
	Head Circumference	2018	512	494	3024 (93.28)
	Gestational Age	2143	570	520	3233 (99.72)
	Small for Gestational Age	1910	554	517	2981 (91.95)
	Large for Gestational Age	1910	554	517	2981 (91.95)
	Preterm	2143	570	521	3234 (99.75)
	Birth Length	2021	537	510	3068 (94.63)
Exposure	Specific Gravity	2107	563	0	2670 (82.35)
	Creatinine	0	0	521	521 (16.07)
	Total Urinary Arsenic	1550	570	514	2634 (81.25)
	Arsenic(III)	1260	0	516	1776 (54.78)
	Arsenic(V)	1260	0	516	1776 (54.78)
	Monomethylarsonic Acid	2107	0	516	2623 (80.91)
	Dimethylarsinic Acid	2107	0	516	2623 (80.91)

**Table 3:** Maternal demographic measures and infant birth outcomes across the three cohorts

	NHBCS		PROTECT		NBCS		Harmonized	
	mean	SD	mean	SD	mean	SD	mean	SD
Maternal BMI (kg/m^2^)	26.25	6.23	26.49	5.37	29.63	7.45	26.64	6.30
Maternal Age (years)	30.81	4.83	27.07	5.54	27.89	6.08	29.72	5.40
Gestational Age (weeks)	38.96	1.78	38.94	1.85	38.66	2.37	38.91	1.90
Birth Length (cm)	50.64	2.91	50.56	2.95	49.99	3.91	50.52	3.12
Birth Weight (g)	3413.51	540.39	3185.41	506.09	3297.36	549.57	3351.28	543.15

**Table 4:** Descriptive statistics of the categorical variables and outcomes of interest

		NBCS	PROTECT	NHBCS
		N (%)		
Preterm Birth	Term	500 (95.97)	526 (92.28)	1961 (91.17)
	Preterm	21 (4.03)	44 (7.72)	182 (8.46)
	Missing	0 (0.0)	0 (0.0)	8 (0.37)
Large for Gestational Age	No	419 (80.42)	500 (87.72)	1444 (67.13)
	Yes	98 (18.81)	54 (9.47)	466 (21.66)
	Missing	4 (0.77)	16 (2.81)	241 (11.2)
Small for Gestational Age	No	491 (94.24)	505 (88.6)	1842 (85.63)
	Yes	26 (4.99)	49 (8.6)	68 (3.16)
	Missing	4 (0.77)	16 (2.81)	241 (11.2)
Education	Less than 11th grade	94 (18.04)	34 (5.96)	18 (0.84)
	High school graduate or equivalent	284 (54.51)	146 (25.61)	199 (9.25)
	Junior college graduate	50 (9.6)	119 (20.88)	313 (14.55)
	College graduate	16 (3.07)	186 (32.63)	695 (32.31)
	Any post-graduate schooling	6 (1.15)	81 (14.21)	567 (26.36)
	Missing	71 (13.63)	4 (0.7)	359 (16.69)
Maternal Race	American Indian or Alaska Native	400 (76.78)	2 (0.35)	7 (0.33)
	Asian	0 (0.0)	0 (0.0)	22 (1.02)
	Black or African American	0 (0.0)	11 (1.93)	6 (0.28)
	Native Hawaiian or Pacific Islander	0 (0.0)	1 (0.18)	0 (0.0)
	White	0 (0.0)	304 (53.33)	1968 (91.49)
	More than one race	35 (6.72)	236 (41.4)	28 (1.3)
	Some other race	0 (0.0)	5 (0.88)	0 (0.0)
	Refused	0 (0.0)	1 (0.18)	1 (0.05)
	Don’t know	0 (0.0)	1 (0.18)	0 (0.0)
	Missing	86 (16.51)	9 (1.58)	119 (5.53)
Sex	Female	261 (50.1)	252 (44.21)	1064 (49.47)
	Male	259 (49.71)	302 (52.98)	1068 (49.65)
	Missing	1 (0.19)	16 (2.81)	19 (0.88)
Smoke during pregnancy	Never smoked	475 (91.17)	485 (85.09)	1620 (75.31)
	Past smoker	27 (5.18)	72 (12.63)	100 (4.65)
	Current smoker	0 (0.0)	0 (0.0)	7 (0.33)
	Smoke during pregnancy	17 (3.26)	7 (1.23)	126 (5.86)
	Missing	2 (0.38)	6 (1.05)	298 (13.85)

**Table 5:** Geometric means and standard deviations of log-transformed concentrations of urinary total arsenic and arsenic species by cohort compared to NHANES

	NHBCS		PROTECT		NBCS		Harmonized		NHANES	
	gMean	gSD	gMean	gSD	gMean	gSD	gMean	gSD	gMean	gSD
Arsenic (III) (μg/L)	0.16	4.15			0.24	2.82	0.18	3.81	0.33	1.06
Arsenic (IV) (μg/L)	0.10	3.79			0.57	1.19	0.16	3.98	0.68	1.03
Dimethylarsinic Acid (μg/L)	2.41	2.75			3.51	2.05	2.59	2.65	3.57	1.03
Monomethylarsonic Acid (μg/L)	0.23	3.34			0.28	2.36	0.24	3.15	0.52	1.05
Total Urinary Arsenic (μg/L)	5.48	3.59	9.66	2.44	4.80	2.44	6.03	3.20	6.80	1.04

**Table 6:** Estimated beta coefficients, corresponding 95% confidence intervals, and confidence interval widths (Models 1.1, 2.1, 3.1)

			NHBCS			PROTECT			NBCS			Harmonized	
Analyte	Outcome		95% CI	CI width		95% CI	CI width		95% CI	CI width		95% CI	CI width
Total Arsenic	Birth Length	0.05	(−0.1,0.19)	0.29	−0.34	(−0.79,0.11)	0.90	0.07	(−0.85,0.99)	1.84	0.01	(−0.13,0.15)	0.28
	Birth Weight	8.64	(−20.7,37.98)	58.68	−59.20	(−129.53,11.13)	140.65	−23.26	(−142.44,95.92)	238.36	−6.50	(−32.74,19.73)	52.47
	Gestational Age	0.01	(−0.07,0.1)	0.17	−0.18	(−0.44,0.08)	0.52	−0.14	(−0.47,0.2)	0.67	−0.03	(−0.1,0.05)	0.16
	Head Circumference	0.00	(−0.08,0.09)	0.16	0.18	(−2.11,2.47)	4.58	−0.36	(−0.91,0.18)	1.09	0.07	(−0.25,0.38)	0.64
Inorganic Arsenic	Birth Length	−0.01	(−0.15,0.12)	0.27				0.32	(−0.42,1.07)	1.49	−0.02	(−0.15,0.11)	0.26
	Birth Weight	−6.34	(−30.27,17.6)	47.87				−54.66	(−151.25,41.94)	193.19	−11.75	(−33.85,10.35)	44.21
	Gestational Age	−0.01	(−0.09,0.07)	0.16				−0.25	(−0.52,0.02)	0.54	−0.01	(−0.09,0.06)	0.15
	Head Circumference	−0.03	(−0.1,0.05)	0.15				0.00	(−0.44,0.45)	0.89	−0.02	(−0.09,0.06)	0.15
Arsenic Sum	Birth Length	0.05	(−0.14,0.25)	0.38				0.17	(−0.75,1.09)	1.84	0.04	(−0.15,0.23)	0.38
	Birth Weight	−8.12	(−44.23,27.99)	72.22				−46.12	(−165.78,73.54)	239.32	−15.17	(−48.92,18.57)	67.49
	Gestational Age	0.02	(−0.09,0.14)	0.23				−0.27	(−0.6,0.06)	0.67	0.01	(−0.09,0.12)	0.21
	Head Circumference	−0.05	(−0.16,0.06)	0.21				−0.32	(−0.86,0.23)	1.09	−0.06	(−0.17,0.05)	0.22

**Table 7:** Estimated beta coefficients, corresponding 95% confidence intervals, and confidence interval widths (Models 1.1, 2.1, 3.1)

			NHBCS			PROTECT			NBCS			Harmonized	
Analyte	Outcome		95% CI	CI width		95% CI	CI width		95% CI	CI width		95% CI	CI width
Total Arsenic	Large for Gestational Age	0.02	(−0.11,0.16)	0.27	−0.20	(−0.69,0.28)	0.97	0.35	(−0.26,0.96)	1.23	0.00	(−0.12,0.13)	0.25
	Preterm Birth	−0.03	(−0.22,0.16)	0.38	0.05	(−0.56,0.67)	1.24	0.92	(−0.3,2.14)	2.44	0.02	(−0.17,0.2)	0.36
	Small for Gestational Age	0.00	(−0.33,0.32)	0.65	0.05	(−0.47,0.57)	1.03	0.20	(−0.72,1.13)	1.85	0.03	(−0.22,0.28)	0.50
Inorganic Arsenic	Large for Gestational Age	0.03	(−0.08,0.14)	0.22				0.02	(−0.48,0.51)	0.99	0.00	(−0.1,0.1)	0.20
	Preterm Birth	0.06	(−0.11,0.23)	0.34				0.81	(−0.2,1.82)	2.02	0.06	(−0.11,0.23)	0.34
	Small for Gestational Age	0.09	(−0.16,0.34)	0.50				−0.21	(−0.98,0.56)	1.54	0.11	(−0.11,0.33)	0.44
Arsenic Sum	Large for Gestational Age	−0.04	(−0.21,0.12)	0.33				0.21	(−0.39,0.82)	1.22	−0.06	(−0.22,0.09)	0.31
	Preterm Birth	−0.12	(−0.37,0.12)	0.49				1.41	(0.09,2.73)	2.64	−0.09	(−0.34,0.15)	0.49
	Small for Gestational Age	0.12	(−0.29,0.52)	0.81				−0.15	(−1.11,0.81)	1.92	0.14	(−0.21,0.49)	0.70

**Table 8:** Random-effects model meta-analysis (models 1.1, 2.1, 3.1)

Analyte	Outcome	Q (*p* value)	*I* ^2^
Total Arsenic	Birth Length	2.6283(0.2687)	35.36%
	Birth Weight	3.1673(0.2052)	43.11%
	Gestational Age	2.4141(0.2991)	30.38%
	Head Circumference	1.6624(0.4355)	20.09%
Inorganic Arsenic	Birth Length	0.7298(0.393)	0.00%
	Birth Weight	0.9057(0.341)	0.00%
	Gestational Age	2.7904(0.095)	64.16%
	Head Circumference	0.0170(0.896)	0.00%
Arsenic Sum	Birth Length	0.0625(0.803)	0.00%
	Birth Weight	0.3551(0.5512)	0.00%
	Gestational Age	2.6455(0.1038)	62.20%
	Head Circumference	0.9060(0.3412)	0.00%

**Table 9:** Random-effects model meta-analysis (models 1.2, 2.2, and 3.2)

Analyte	Outcome	Q (*p* value)	*I* ^2^
Total Arsenic	Large for Gestational Age	1.9146(0.3839)	0.00%
	Preterm Birth	2.3049(0.3159)	0.00%
	Small for Gestational Age	0.1681(0.9194)	0.00%
Inorganic Arsenic	Large for Gestational Age	0.0015(0.9692)	0.00%
	Preterm Birth	2.0600(0.1512)	51.46%
	Small for Gestational Age	0.5275(0.4676)	0.00%
Arsenic Sum	Large for Gestational Age	0.6106(0.4346)	0.00%
	Preterm Birth*	4.9893(0.0255)	79.96%
	Small for Gestational Age	0.2580(0.6115)	0.00%
