# Supplementary material for: Effects of maternal arsenic exposure on birth outcomes using harmonized data across three birth cohorts
Source: Toxicol Environ Health Sci. 2026 Jan 29;18(2):257–69. doi: 10.1007/s13530-025-00292-6 (PMC12928382; doi:10.1007/s13530-025-00292-6)
Supplement: Supplementary file 1 — Supplementary file1 (DOCX 34 kb) [file 13530_2025_292_MOESM1_ESM.docx]

Table S1. Data harmonization concordance summary

| Variable | Metric | NBCS | N (NBCS) | NHBCS | N (NHBCS) | PROTECT | N (PROTECT) | All | N (All) | % Difference | Test | Test Statistic |
| --- | --- | --- | --- | --- | --- | --- | --- | --- | --- | --- | --- | --- |
| Sex | % | 49.8 | 520 | 50.1 | 2132 | 54.5 | 554 | 50.8 | 3206 | 9.1 | Chi-square | X2=3.685, p=0.158, df=2 |
| Maternal Age (years) | Med. [IQR] | 27.2 [23.3-32.0] | 451 | 31.0 [28.0-34.0] | 2151 | 27.0 [23.0-31.0] | 569 | 30.0 [26.0-33.2] | 3171 | 14.1 | AD | A2=144.916, p=0.001 |
| Smoke During Pregnancy | % | 15 | 519 | 26.6 | 1853 | 16.5 | 564 | 22.6 | 2936 | 59.9 | Chi-square | X2=72.399, p=<0.001, df=6 |
| Maternal BMI | Med. [IQR] | 28.7 [25.3-33.3] | 298 | 24.6 [21.9-28.9] | 2078 | 25.8 [22.7-29.7] | 538 | 25.2 [22.2-29.7] | 2914 | 15.6 | AD | A2=44.979, p=0.001 |
| Parity | Med. [IQR] | 1.0 [0.0-3.0] | 521 | 1.0 [0.0-1.0] | 2087 | 0.0 [0.0-1.0] | 419 | 1.0 [0.0-1.0] | 3027 | 150 | AD | A2=103.726, p=0.001 |
| Maternal Educational Attainment | Mode | 2 | 450 | 4 | 1792 | 4 | 566 | 4 | 2808 |  | Chi-square | X2=1033.261, p=<0.001, df=8 |
| Birth Weight | Med. [IQR] | 3340.7 [2980.1-3646.6] | 520 | 3430.3 [3125.0-3741.5] | 1930 | 3200.0 [2900.0-3500.0] | 555 | 3375.0 [3047.0-3692.2] | 3005 | 6.9 | AD | A2=44.764, p=0.001 |
| Head Circumference | Med. [IQR] | 34.5 [33.5-35.5] | 494 | 34.5 [33.5-35.5] | 2018 | 34.0 [33.0-34.9] | 512 | 34.5 [33.5-35.5] | 3024 | 1.5 | AD | A2=47.824, p=0.001 |
| Gestational Age | Med. [IQR] | 39.0 [38.0-40.0] | 520 | 39.0 [38.3-40.0] | 2143 | 39.3 [38.3-40.0] | 570 | 39.0 [38.1-40.0] | 3233 | 0.7 | AD | A2=8.787, p=0.001 |
| Large for Gestational Age | % | 19 | 517 | 24.4 | 1910 | 9.7 | 554 | 20.7 | 2981 | 83.1 | Chi-square | X2=57.290, p=<0.001, df=2 |
| Small for Gestational Age | % | 5 | 517 | 3.6 | 1910 | 8.8 | 554 | 4.8 | 2981 | 89.7 | Chi-square | X2=26.334, p=<0.001, df=2 |
| Preterm Birth | % | 4 | 521 | 8.5 | 2143 | 7.7 | 570 | 7.6 | 3234 | 66.8 | Chi-square | X2=11.835, p=0.00269, df=2 |
| Birth Length | Med. [IQR] | 50.6 [48.7-52.0] | 510 | 50.8 [49.0-52.5] | 2021 | 50.8 [49.5-52.1] | 537 | 50.8 [49.0-52.1] | 3068 | 0.4 | AD | A2=6.721, p=0.001 |
| Total Urinary Arsenic | Med. [IQR] | 5.2 [3.7-7.5] | 514 | 5.7 [3.5-11.1] | 1549 | 10.6 [6.7-16.0] | 563 | 6.4 [3.9-11.7] | 2626 | 76.4 |  | A2=131.827, p=0.001 |
| Dimethylarsinic Acid | Med. [IQR] | 4.0 [2.8-5.8] | 516 | 2.7 [1.8-4.3] | 2063 |  | 0 | 2.9 [2.0-4.7] | 2579 | 38.6 | AD | A2=90.293, p=0.001 |
| Monomethylarsonic Acid | Med. [IQR] | 0.3 [0.2-0.6] | 516 | 0.3 [0.2-0.5] | 2063 |  | 0 | 0.3 [0.2-0.5] | 2579 | 19.3 | AD | A2=8.816, p=0.001 |
| Arseinc(III) | Med. [IQR] | 0.3 [0.1-0.5] | 516 | 0.2 [0.1-0.4] | 1216 |  | 0 | 0.2 [0.1-0.5] | 1732 | 39.2 | AD | A2=38.712, p=0.001 |
| Arsenic(V) | Med. [IQR] | 0.6 [0.4-1.1] | 516 | 0.1 [0.0-0.3] | 1216 |  | 0 | 0.2 [0.1-0.6] | 1732 | 144 | AD | A2=361.673, p=0.001 |

Table S2. Estimated beta coefficients, corresponding 95% confidence intervals and confidence interval widths for models 1.1, 2.1, and 3.1 with infant sex as an effect modifier

|  |  | NHBCS | | | PROTECT | | | NBCS | | | Harmonized | | |
| --- | --- | --- | --- | --- | --- | --- | --- | --- | --- | --- | --- | --- | --- |
|  | Outcome | β | 95% CI | CI width | β | 95% CI | CI width | β | 95% CI | CI width | β | 95% CI | CI width |
| Total Arsenic: Sex=Male | Birth Length | -0.12 | (-0.41,0.17) | 0.58 | -0.96 | -1.86,-0.06 | 1.8 | 0.05 | (-1.78,1.89) | 3.67 | -0.19 | (-0.47,0.09) | 0.56 |
|  | Birth Weight | 24.65 | (-33.29,82.6) | 115.89 | -170.47 | -310.98,-29.96 | 281.02 | 100.79 | (-136.24,337.82) | 474.06 | -8.88 | (-59.82,42.06) | 101.88 |
|  | Gestational Age | 0 | (-0.17,0.17) | 0.34 | -0.4 | -0.93,0.12 | 1.05 | 0.24 | (-0.43,0.90) | 1.33 | -0.01 | (-0.17,0.14) | 0.3 |
|  | Head Circumference | -0.03 | (-0.19,0.13) | 0.33 | -0.83 | -5.49,3.82 | 9.3 | 0.99 | (-0.09,2.07) | 2.16 | 0.06 | (-0.57,0.69) | 1.26 |
| Inorganic Arsenic: Sex=Male | Birth Length | 0.01 | (-0.26,0.27) | 0.53 |  |  |  | -0.02 | (-1.47,1.44) | 2.91 | -0.05 | (-0.30,0.21) | 0.51 |
|  | Birth Weight | 14.91 | (-32.79,62.61) | 95.4 |  |  |  | -56.83 | (-245.88,132.21) | 378.09 | 21.36 | (-21.90,64.62) | 86.52 |
|  | Gestational Age | 0.06 | (-0.09,0.22) | 0.31 |  |  |  | -0.46 | (-0.98,0.07) | 1.05 | -0.02 | (-0.16,0.12) | 0.28 |
|  | Head Circumference | -0.07 | (-0.22,0.07) | 0.3 |  |  |  | 0.27 | (-0.60,1.15) | 1.75 | -0.03 | (-0.18,0.11) | 0.29 |
| Arsenic Sum: Sex = Male | Birth Length | 0.15 | (-0.24,0.53) | 0.76 |  |  |  | -0.15 | (-1.97,1.67) | 3.63 | 0.09 | (-0.29,0.47) | 0.76 |
|  | Birth Weight | 39.86 | (-32.02,111.73) | 143.75 |  |  |  | -1.72 | (-237.30,233.87) | 471.17 | 45.02 | (-22.47,112.50) | 134.97 |
|  | Gestational Age | 0.03 | (-0.19,0.26) | 0.45 |  |  |  | -0.31 | (-0.96,0.35) | 1.31 | -0.04 | (-0.24,0.17) | 0.41 |
|  | Head Circumference | 0.05 | (-0.17,0.26) | 0.43 |  |  |  | 1.07 | (-0.00,2.14) | 2.14 | 0.13 | (-0.08,0.35) | 0.43 |

Table S3. Estimated beta coefficients, corresponding 95% confidence intervals and confidence interval widths for models 1.1, 2.1, and 3.1 with infant sex as an effect modifier

|  |  | NHBCS | | | PROTECT | | | NBCS | | | Harmonized | | |
| --- | --- | --- | --- | --- | --- | --- | --- | --- | --- | --- | --- | --- | --- |
|  | Outcome | β | 95% CI | CI width | β | 95% CI | CI width | β | 95% CI | CI width | β | 95% CI | CI width |
| Total Arsenic: Sex=Male | Large for Gestational Age | 0.05 | (-0.22,0.32) | 0.55 | -0.42 | (-1.41,0.57) | 1.98 | 0.4 | (-0.92,1.71) | 2.63 | 0 | (-0.25,0.25) | 0.49 |
|  | Preterm Birth | 0.02 | (-0.35,0.40) | 0.75 | 1.31 | (0.07,2.56) | 2.48 | 0.03 | (-2.51,2.57) | 5.08 | 0.11 | (-0.24,0.47) | 0.71 |
|  | Small for Gestational Age | -0.43 | (-1.14,0.27) | 1.41 | 0.77 | (-0.38,1.92) | 2.31 | -1.94 | (-4.27,0.40) | 4.66 | -0.05 | (-0.56,0.46) | 1.01 |
| Inorganic Arsenic: Sex=Male | Large for Gestational Age | -0.03 | (-0.25,0.20) | 0.45 |  |  |  | 0.4 | (-0.74,1.55) | 2.29 | 0.2 | (-0.00,0.40) | 0.4 |
|  | Preterm Birth | -0.24 | (-0.58,0.10) | 0.68 |  |  |  | 0.21 | (-1.90,2.33) | 4.23 | -0.19 | (-0.51,0.14) | 0.64 |
|  | Small for Gestational Age | -0.63 | (-1.15,-0.11) | 1.04 |  |  |  | -1.35 | (-3.16,0.46) | 3.62 | -0.7 | (-1.14,-0.26) | 0.88 |
| Arsenic Sum: Sex = Male | Large for Gestational Age | 0.03 | (-0.32,0.37) | 0.69 |  |  |  | 0.84 | (-0.58,2.25) | 2.83 | 0.26 | (-0.05,0.58) | 0.63 |
|  | Preterm Birth | -0.15 | (-0.64,0.34) | 0.98 |  |  |  | 0.63 | (-2.05,3.32) | 5.36 | -0.06 | (-0.54,0.41) | 0.95 |
|  | Small for Gestational Age | -0.83 | (-1.65,-0.02) | 1.63 |  |  |  | -1.49 | (-3.60,0.63) | 4.23 | -0.97 | (-1.67,-0.26) | 1.41 |
